# A content analysis of the reliability and quality of Youtube videos as a source of information on health-related post-COVID pain

**DOI:** 10.7717/peerj.14089

**Published:** 2022-09-28

**Authors:** Erkan Ozduran, Sibel Büyükçoban

**Affiliations:** 1 Physical Medicine and Rehabilitation, Algology, Dokuz Eylul University, Izmir, Türkiye; 2 Anesthesiology and Reanimation, Dokuz Eylül University, Izmir, Türkiye

**Keywords:** COVID-19, E-learning, Pain, Post-acute COVID-19 syndrome, YouTube

## Abstract

**Background:**

The use of the internet as a source of information has increased during the pandemic, and YouTube has become an increasingly important source of information on Coronavirus disease 2019 (COVID-19). In the long COVID picture, which occurs when symptoms related to COVID-19 last longer than 1 month, pain involving the musculoskeletal system affects the quality of life quite negatively. The aim of this study was to investigate the informational value and quality of YouTube videos related to post-COVID pain.

**Methods:**

In this study, 180 videos were listed using the search terms “pain after COVID,” “post-COVID pain,” and “long COVID and pain”(15 April 2022). Videos were classified according to video parameters and content analysis. Quality, reliability and accuracy of the videos were determined with the Global Quality Score (GQS), the Journal of American Medical Association (JAMA) Benchmark Criteria and the Modified DISCERN Questionnaire, respectively.

**Results:**

One hundred videos that met the inclusion criteria were included in the assessment. Of these videos, 74 were found to be of low quality, 14 of moderate quality, and 12 of high quality; 21% contained insufficient data, 73% contained partially sufficient data, and 6% contained completely sufficient data. Videos uploaded by academic sources (66.7%) and physicians (12.5%) made up the majority of the high-quality group. A statistically significant correlation was found between the source of upload and number of views (*p* = 0.014), likes (*p* = 0.030), comments (*p* = 0.007), and video duration (*p* = 0.004). Video duration was found to have a poor positive correlation with GQS (r = 0.500), JAMA (r = 0.528), and modified DISCERN (r = 0.470) scores (*p* < 0.001).

**Conclusion:**

The findings of this study revealed that the majority of YouTube videos on post-COVID pain had low quality and partially sufficient data. High-quality videos were found to have longer durations and were uploaded by academic sources and physicians. The fact that only videos with English content at a certain time can be counted among the limitations. For patients suffering from post-COVID pain whose access to healthcare services was interrupted during the COVID pandemic, YouTube can be considered as an alternative source as well as a means of telerehabilitation. It can be argued that higher quality videos created by healthcare professionals could aid in patient education in the future.

## Introduction

Coronavirus disease 2019 (COVID-19), which was first detected in late December 2019, was declared a pandemic by the World Health Organization (WHO) on March 11, 2020 and became the first coronavirus pandemic of the 21th century (Andika et al., 2021). To date, 500 million cases and more than six million deaths have been reported globally, according to WHO data for April 2022 (WHO Team, 2022). The clinical manifestations of the disease have a wide spectrum, ranging from the respiratory system to the musculoskeletal system and from moderate symptoms to severe organ damage. Fever (77.4–98.6%) and cough (59.4–81.8%) are the two main clinical symptoms, and other common symptoms include fatigue (38.1–69.6%), myalgia (11.1–34.8%), and headache (6.5–33.9%) (Ge et al., 2020).

There are reports that myalgia, chest pain, and headache occur at varying rates in COVID-19, but initial symptoms of the disease are usually myalgia and headache (Tang, Comish & Kang, 2020). The pathogenesis of pain during COVID-19 disease is clearly multifactorial, and its cause is still under investigation. Extensive tissue and organ damage caused by infection, damage to tissues such as muscles and joints, and increased cytokines have been shown to be involved in the pathogenesis of pain. Therefore, pain is both a leading symptom of the disease and a significant cause of disease-related disability (Şahin et al., 2021).

COVID-19 symptoms can last for more than a month in some cases, which is referred to as post-COVID, long COVID, or prolonged COVID (Nabavi, 2020). According to Şahin et al. (2021), pain symptoms can last for 7 to 78 days after COVID-19 infection. There is no agreement on how to diagnose and treat cases of prolonged symptoms (Greenhalgh et al., 2020). It is important to remember that post-COVID pain, which is a significant part of the disease’s clinical manifestations, can prevent individuals from returning to their normal lives and performing daily functions after the acute period of the disease (Şahin et al., 2021).

During the pandemic, when patients stayed home and were afraid to visit hospitals, the internet became an important means of accessing information. A considerable number of internet users have sought information about their symptoms and treatments for these symptoms on YouTube, the second most visited website after Google (Chan et al., 2021). This popular video-sharing platform with more than two billion monthly visitors has recorded an increase of 75% in the view rates of news and health-related videos compared to the pre-pandemic period (Andika et al., 2021). Although it has several advantages, such as being free, easy to access, and having an extensive network of information, it can also cause irreversible problems for COVID-19 survivors due to potentially misleading informational content (Tolu et al., 2018).

Not only the research on health-related issues on youtube, but also the changes in many layers of the digital platform during the pandemic process were observed. An example of this is that people turn to applications that order food while they stay at home and gain the habit of paying money in the digital environment (Chaveesuk, Khalid & Chaiyasoonthorn, 2021; Muangmee et al., 2021). These transformations in digital life have caused us to gain a different lifestyle compared to the past, and as a result, health problems have arisen by leaving the active life and also brought security problems related to digital use.

There are numerous studies that have evaluated YouTube videos on COVID-19 from different perspectives (Andika et al., 2021; Jacques et al., 2022; Li et al., 2020). However, to the best of our knowledge, no study has yet analyzed health related post-COVID pain, which is a significant clinical manifestation of the disease and a cause of disability. Therefore, the primary goal of this study was to evaluate YouTube videos on post-COVID pain in terms of reliability and quality. We also aimed to identify high-quality and reliable video upload sources. Finally, another aim was to compare user parameters for videos with high, moderate, and low quality and reliability.

## Materials and Methods

### Ethical approval

The Dokuz Eylul University ethics committee approval was obtained (Ethics Committee decision no: 6957-GOA 2022/06-08, Date: 16.02.2022) for this descriptive content analysis study.

### Content analysis

A video search was conducted on YouTube on April 15, 2022 using the keywords “pain after COVID,” “post-COVID pain,” and “long COVID and pain” on the YouTube search engine (https://www.youtube.com). Two authors worked together to obtain the most comprehensive listing of relevant videos and determined the three search terms by consensus. Previous studies have shown that a significant number of internet users only view search results listed on the first three pages (60 videos in total, 20 on each page) (Onder & Zengin, 2021; Rittberg, Dissanayake & Katz, 2016). Although YouTube has switched from page ranking to continuous listing, we based our search on the method used in similar studies and identified 60 videos for each search term (Kocyigit & Akyol, 2021; Tolu et al., 2018). Video listing was done based on the number of views to evaluate videos with the greatest impact on society. To avoid bias based on search history and cookies, the authors used Google Chrome’s incognito mode. A total of 180 videos were identified, and the evaluation was conducted by the researchers (EO and SB) who were blind to each other’s evaluation results. Videos were included in the evaluation if they were in English language, had audio, and were relevant to the subject. Duplicate videos, non-English-language videos, and videos with audio–video problems were excluded (Kocyigit & Akyol, 2021).

### Content categories

The content categories of the videos was evaluated by the presence/absence of the following eight factors related to post-COVID pain in each video: (1) Etiology, (2) Diagnosis, (3) Non-pain symptoms, (4) Treatment, (5) Exercise, (6) Prevention, (7) Risk Factors, and (8) Vaccine–pain relationship.

### Assesment of quality

Developed by Bernard et al. (2007), GQS is a five-point Likert scale that indicates the quality, ease of use, and flow of websites. Scoring according to this scale was as follows: one point; poor quality, two points; generally poor quality and poor flow, three points; moderate quality, suboptimal flow, four points; good quality and generally good flow, five points, excellent quality and excellent flow, very useful for patients (Table 1). According to the score, the videos are divided into three quality groups. Videos with a score of 4 and 5 are considered high quality, videos with a score of 3 are considered intermediate quality, and videos with a score of 1 and 2 are considered low quality. If the scores of the two authors did not match, a third researcher (VH) conducted the evaluation and his scoring was determined as the final score (Kocyigit & Akyol, 2021).

**Table 1:** Contents of JAMA, DISCERN and GQS assessment criteria.

JAMA benchmark criteria	Total score (0–4 points)
Authorship	1 Point (Authors and contributors, their affiliations, and relevant credentials should be provided)
Attribution	1 Point (References and sources for all content should be listed)
Disclosure	1 Point (Conflicts of interest, funding,sponsorship, advertising, support, and video ownership should be fully disclosed)
Currency	1 Point (Dates that on which the content was posted and updated should be indicated). JAMA is used to evaluate the accuracy and reliability of information)
	
**DISCERN criteria**	**Total score (16–80 points)**
1 Are the aims clear?	1–5 point
2 Does it achieve its aims?	1–5 point
3 Is it relevant?	1–5 point
4 Is it clear what sources of information were used	1–5 point
5 Is it clear when the information used or reported in the publication was produced?	1–5 point
6 Is it balanced and unbiased?	1–5 point
7 Does it provide details of additional sources of 1.45 support and information?	1–5 point
8 Does it refer to areas of uncertanity?	1–5 point
9 Does it describe how each treatment works?	1–5 point
10 Does it describe the benefits of each treatment?	1–5 point
11 Does it describe the risks of each treatment?	1–5 point
12 Does it describe what would happen if no treatment is used?	1–5 point
13 Does it describe how the treatment choices affect overall quality of life?	1–5 point
14 Is it clear that there may be more than one possible treatment choice?	1–5 point
15 Does it provide support for shared decision making?	1–5 point
16 Based on the answers to all of the above questions, rate the overall quality of the publication as a source of information about treatment choices.	1–5 point
**GQS**	**Score**
Poor quality, poor flow of the site, most information missing, not at all useful for patients	1
Generally poor quality and poor flow, some information listed but many important topics missing, of very limited use to patients	2
Moderate quality, suboptimal flow, some important information is adequately discussed but others poorly discussed, somewhat useful for patients	3
Good quality and generally good flow, most of the relevant information is listed, but some topics not covered, useful for patients	4
Excellent quality and excellent flow, very useful for patients	5

**Note:**

JAMA, Journal of American Medical Association; GQS, Global Quality Score.

Another scale used in quality assessment is the modified DISCERN scale. It is a scoring tool consisting of yes/no questions rated on a 5-point scale designed to evaluate the quality and reliability of written health information. It can be scored from 0 to 5 points, and the total score is obtained by the sum of “yes” points (yes = 1 point and no = 0 point). The questions included in the questionnaire are: “Does the video address areas of controversy/uncertainty?”, “Are additional sources of information listed for patient reference?”, “Is the provided information balanced and unbiased?”, “Are valid sources cited?” (from valid studies, physiatrists),” “Is the video clear, concise, and understandable” (Charnock et al., 1999) (Table 1).

### Assesment of reliability

The Journal of American Medical Association (JAMA) benchmark criteria were used to review online videos and resources based on four criteria: authorship, attribution, disclosure, and currency. JAMA is used to evaluate the reliability and accuracy of videos (Table 1). Each met criterion receives one point, with a maximum score of 0–4. A score of four points indicates highest quality (Silberg, Lundberg & Musacchio, 1997). Videos with a score of 0–1 point have insufficient data content, videos with a score of 2–3 have partially sufficient data content, and videos with a score of 4 have completely sufficient data content.

### Evaluating the user engagement (video parameters)

Four interaction measurements recorded for each video; (1) views, (2) likes, (3) video duration (seconds) and (4) comments. It is known that Youtube has not shown the dislike button to its users recently and therefore the dislike averages are low. For this reason, dislike numbers were not taken into account in this study.

### Video sources

Video sources were categorized as follows: academic, physician, society/professional organization, health-related websites, patients, and news channels. Other collected data included the presence/absence of animated content and the country/continent of origin.

### Statistical analysis

Acquired data were analyzed using the SPSS (Statistical Package for Social Sciences, Chicago, IL, USA) 24.0 software. Continuous variables were expressed as mean ± standard deviation, while frequency data were expressed as numbers (*n*) and percentages (%). Kolmogorov–Smirnov and Shapiro–Wilk tests were used to check whether continuous datasets were normally distributed. Kruskal–Wallis and Mann–Whitney U tests were used to analyze continuous data sets depending on the number of groups. Frequency data were analyzed using Pearson’s chi-square test. Correlation analysis was performed using Pearson correlation testing to compare the groups. The significance level was set at *p* < 0.05.

## Results

**Figure 1:**
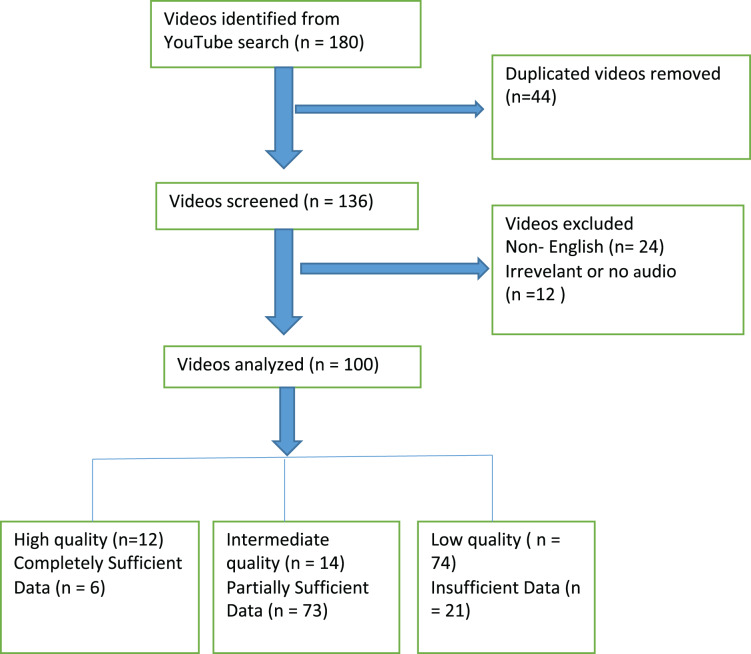
Flow diagram for review of Youtube videos on post-COVID pain.

Initially, 180 videos were listed, and duplicate videos were identified. One of the duplicate videos was included in the evaluation, and 44 videos were excluded. The remaining 136 videos were screened based on the exclusion criteria; irrelevant and non-English-language videos were excluded, and finally, 100 videos that met the inclusion criteria were evaluated (Fig. 1). A total of 19 h and 6 s of video were watched. The longest video lasted 1 h, 21 min, and 34 s, while the shortest lasted 45 s. The most-liked video had 10,000 likes, and the least-liked video had no likes. The most-viewed video had been viewed 1,296,088 times, and the least-viewed video had been viewed six times. The video with the highest number of comments had 3,584 comments, and the video with the fewest comments had none. The average number of views per video was 58,429.91 ± 155,523.6, the average number of likes was 660.08 ± 1,761.94, the average number of comments was 198.12 ± 485.11, and the average video duration was 684.06 ± 886.69 s. There were 22 (22%) animated videos. Most of the videos (42 [42%] videos) were uploaded in 2021. A statistically significant difference was found between the videos with animation and diagnosis on the basis of years. No significant correlation was found between the JAMA, DISCERN and GQS results of the videos by years (Tables 2 and 3).

**Table 2:** Comparison of the content of videos over the years.

Video content/years		2020, *n* (%)	2021, *n* (%)	2022, *n* (%)	*p*
Animation	+	1 (3.2%)	12 (28.6%)	9 (33.8%)	**0.009**
	−	30 (96.8%)	30 (71.4%)	18(66.7%)	
Etiology	+	10 (32.3%)	21 (50%)	14 (51.9%)	0.281
	–	21 (67.7%)	21 (50%)	13 (48.1%)	
Diagnose	+	11 (35.5%)	18 (42.9%)	3 (11.1%)	**0.020**
	–	20 (64.5%)	24 (57.1%)	24 (88.9%)	
Non-pain symptom	+	20 (64.5%)	31 (73.8%)	21 (77.8%)	0.502
	–	11 (35.5%)	11 (26.2%)	6 (22.2%)	
Treatment	+	24 (77.4%)	32 (76.2%)	23 (82.5%)	0.648
	–	7 (22.6%)	10 (23.8%)	4 (14.8%)	
Exercise	+	15 (48.4%)	19 (45.2%)	14 (51.9%)	0.865
	–	16 (51.6%)	23 (54.8%)	12 (48.1%)	
Prevention	+	12 (38.7%)	17 (40.5%)	9 (33.3%)	0.833
	–	19 (61.3%)	25 (59.5%)	18 (66.7%)	
Risk factors	+	5 (16.1%)	7 (16.7%)	5 (18.5%)	0.968
	–	26 (83.9%)	35 (83.3%)	22 (81.5%)	
Vaccine-pain relationship	+	0 (0%)	3 (7.1%)	2 (7.4%)	0.306
	–	31 (100%)	39 (92.9%)	25 (92.6%)	
JAMA	Insufficient data (1 Point)	11 (35.5%)	7 (16.7%)	3 (11.1%)	0.176
	Partially sufficient data (2 or 3 points)	18 (58.1%)	33 (78.6%)	22 (81.5%)	
	Completely sufficient data (4 points)	2 (6.5%)	2 (4.8%)	2 (7.4%)	
GQS	Low quality (1 or 2 points)	22 (71%)	32 (76.2%)	20 (74.1%)	0.946
	Intermediate quality (3 points)	5 (16.1%)	6 (14.3%)	3 (11.1%)	
	High quality (4–5 points)	4 (12.9%)	4 (9.5)	4 (14.8%)	
Modified DISCERN	1 Point	8 (25.8%)	7 (16.7%)	3 (11.1%)	0.293
	2 Points	8 (25.8%)	18 (42.9%)	15 (55.6%)	
	3 Points	11 (35.5%)	11 (26.2%)	4 (14.8%)	
	4 Points	4 (12.9%)	4 (9.5%)	3 (11.1%)	
	5 Points	0 (0%)	2 (4.8%)	2 (7.4%)	

**Note:**

Pearson Chi-square test. GQS, Global Quality Score; JAMA, Journal of American Medical Association benchmark criteria, Bold font: statistical significance.

**Table 3:** Video characteristics according to years and assesment parameters (mean ± standard deviation).

Years	Follow-up Mean ± SD	Like Mean ± SD	Comment Mean ± SD	Time Mean ± SD
2020 (*n* = 31)	102,026.19 ± 249,481.14	824.67 ± 2,060.31	365.51 ± 773.86	504.16 ± 566.921
2021 (*n* = 42)	44,484.73 ± 92,455.02	432.14 ± 1,245.57	106.47 ± 218.46	728.81 **± **979.127
2022 (*n* = 27)	30,067.40 **± **58,200.16	825.66 ± 2,081.25	148.48 ± 283.33	821 **± **1,024.604
***p***	**0.014**	0.413	0.164	0.098
**Video sources**				
Academic (*n* = 8)	23,100.25 ± 52,158.631	618.12 ± 1,530.28	112 ± 270.77	2,052.25 ± 1,675.03
Physician (*n* = 8)	34,109.12 ± 43,279.41	462.50 ± 523.64	312.50 ± 505.21	1,014.50 ± 1,271.617
Society/Professional Organization (*n* = 16)	34,368.56 ± 77,429.74	180.37 ± 286.7	45.81 ± 81.87	580.69 ± 805.682
Health-related Website (*n* = 33)	56,987.78 ± 106,251.83	818.57 ± 2,293.62	144.66 ± 358.19	569.67 ± 688.075
Patient (*n* = 7)	2,502.85 ± 4,955.69	58.85 ± 109.59	27.57 ± 41.23	601.57 ± 303.345
News (*n* = 28)	104,903.64 ± 258,532.67	966.14 ± 2,012.83	382.71 ± 745.21	413.25 ± 326.92
***p***	**0.014**	**0.030**	**0.007**	**0.004**
**GQS (1–5 points)**				
Low quality (1 or 2 points) (*n* = 74)	59,734.91 ± 171,660.96	528.04 ± 1,417.22	196.01 ± 518.03	503.32 ± 616.907
Intermediate quality (3 points) (*n* = 14)	47,206.42 ± 66,972.39	895.57 ± 2,624.98	185.07 ± 310.41	642 ± 723.287
High quality (4–5 points) (*n* = 12)	63,476.41 ± 128,961.16	1,199.58 ± 2,441.74	226.33 ± 471.23	1,847.67 ± 1,494.37
***p***	0.539	0.635	0.467	**0.001>**
**JAMA score (0–4 Points)**				
Insufficient data (1 Point) (*n* = 21)	142,488.85 ± 297,662.14	1,205 ± 2,458.78	410.14 ± 891.16	325.24 ± 250.686
Partially sufficient data (2 or 3 points) (*n* = 73)	32,846.91 ± 61,027.50	448.10 ± 1,312.31	149.15 ± 291.17	611.9 ± 696.5
Completely sufficient data (4 points) (*n* = 6)	75,483.33 ± 176,893.24	1,331.83 ± 3,168.89	51.83 ± 111.94	2,817.83 ± 1,500.016
***p***	0.018	0.098	0.319	**0.001>**
**Modified DISCERN score (0–5 points)**				
1 Points (*n* = 18)	145,217 ± 318,483.29	1,073.5 ± 2,455.12	447.05 ± 901.89	339.83 ± 234.648
2 Points (*n* = 41)	37,995.95 ± 77,567.24	437.51 ± 944.61	131.29 ± 318.69	547.49 ± 663.42
3 Points (*n* = 26)	34,248.03 ± 52,953.52	539.46 ± 1,935.72	136.76 ± 242.97	609.19 ± 780.73
4 Points (*n* = 11)	31,076.27 ± 55,168.29	624.18 ± 1,333.74	230.09 ± 490.41	974.09 ± 651.54
5 Points (*n* = 4)	109,740.75 ± 217,844.64	19,6375 ± 3,890.88	73.75 ± 137.61	3,322 ± 1,633.85
***p***	0.074	0.484	0.059	**0.001>**

**Note:**

Mann Whitney U test. N, Number of videos; SD, Standart Deviation; GQS, Global Quality Score; JAMA, Journal of American Medical Association benchmark criteria, Bold font: statistical significance (*p* < 0.05).

According to the content categories of the post-COVID pain videos, 45 (45%) contained information on etiology, 32 (32%) on diagnosis, 72 (72%) on non-pain symptoms, 79 (79%) on treatment, 48 (48%) on exercise, 38 (38%) on risk factors, and 5 (5%) on vaccine–pain relationship.

According to the GQS results, 74 of the videos were of low quality, 14 were of moderate quality, and 12 were of high quality. High-quality videos had mostly been uploaded by academic sources (66.7%), followed by professional societies/organizations (16.7%). The source of upload had a statistically significant correlation with GQS, modified DISCERN, and JAMA scores for quality and reliability (*p* < 0.001). News videos had more views, likes, and comments. Videos uploaded by academic sources had longer durations. There was a statistically significant difference between the upload source and number of views (*p* = 0.014), likes (*p* = 0.030), comments (*p* = 0.007), and duration (*p* = 0.004). This statistical difference can be explained by the higher number of views, likes, and comments on news videos and the longer duration of videos uploaded by academic sources (Table 4).

**Table 4:** Video sources by assesment parameters.

		Academic (*n*)	Physician (*n*)	Society/Professional Organization (*n*)	Health-related Website (*n*)	Patient (*n*)	News (*n*)	*p*
GQS (1–5 points)	Low quality (1or 2 points)	0 (0%)	6 (75%)	9 (56.3%)	24 (72.7%)	7(100%)	28 (100%)	**>0.001**
	Intermediate quality (3 points)	0 (0%)	1 (12.5%)	5 (31.3%)	8 (24.2%)	0(0%)	0 (0%)	
	High quality (4–5 points)	8 (100%)	1 (12.5%)	2 (12.5%)	1 (3%)	0(0%)	0 (0%)	
JAMA score (0–4 points)	Insufficient data (1 point)	0 (0%)	0 (0%)	0 (0%)	6 (18.2%)	2(28.6%)	13 (46.4%)	**>0.001**
	Partially sufficient data (2 or 3 points)	4 (50%)	8 (100%)	15 (93.8%)	26 (78.8%)	5 (71.4%)	15 (53.6%)	
	Completely sufficient data (4 points)	4 (50%)	0 (0%)	1 (6.3%)	1 (3%)	0 (0%)	0 (0%)	
Modified DISCERN score (0–5 points)	1 Point	0 (0%)	0 (0%)	0 (0%)	3 (9.1%)	1 (14.3%)	14 (50%)	**>0.001**
	2 Points	0 (0%)	4 (50%)	5 (31.3%)	15 (45.5%)	5 (71.4%)	12 (42.9%)	
	3 Points	0 (0%)	2 (25%)	9 (56.3%)	12 (36.4%)	1 (14.3%)	2 (7.1%)	
	4 Points	5 (62.5%)	2 (25%)	2 (12.5%)	2 (6.1%)	0 (0%)	0 (0%)	
	5 Points	3 (37.5%)	0 (0%)	0 (0%)	1 (3%)	0 (0%)	0 (0%)	

**Notes:**

Pearson Chi-square test. GQS, Global Quality Score, JAMA, Journal of American Medical Association benchmark criteria.

Bold indicates statistical significance.

There was a weak positive correlation among JAMA (r = 0.528, *p* < 0.001), modified DISCERN (r = 0.470, *p* < 0.001), and GQS (r = 0.500, *p* < 0.001) scores. Videos with higher quality and credibility were found to have a longer duration (Table 5). The same was applicable to the correlation between the source of upload and duration (*p* < 0.001). Videos uploaded by academic sources and physicians had a longer duration (Table 3).

**Table 5:** Correlations between quantitative variables and scores.

	GQS		JAMA		Modified DISCERN	
	r	*p*	r	*p*	r	*p*
Number of views	−0.126	0.213	−0.135	0.180	−0.137	0.175
Number of likes	0.041	0.686	0.058	0.565	0.024	0.809
Number of comments	−0.096	0.340	−0.142	0.160	−0.142	0.160
Video duration; second	0.500*	**>0.001**	0.528*	**>0.001**	0.470*	**>0.001**
GQS	–	**-**	0.857*	**>0.001**	0.888*	**>0.001**
JAMA	0.857*	**>0.001**	–	**-**	0.843*	**>0.001**
Modified DISCERN	0.888*	**>0.001**	0.843*	**>0.001**	–	–

**Notes:**

Pearson correlation test. GQS, Global Quality Score; JAMA, Journal of American Medical Association benchmark criteria.

Bold indicates statistical significance.

* Significant correlation.

The continent of origin had a statistically significant correlation with the number of comments (*p* = 0.039) but not with video duration. This statistical difference can be explained by the fact that videos originating from the American continent had more comments. There was no statistically significant correlation between the country of origin and video characteristics (views *p* = 0.439, likes *p* = 0.436, comments *p* = 0.066, and duration *p* = 0.361) (Table 6).

**Table 6:** Evaluation of user parameters by continent and country.

	View Mean ± SD	Like Mean ± SD	Comment Mean ± SD	Time Mean ± SD
**Continent**				
America (*n* = 63)	69,896.2 ± 186,996.89	902.47 ± 2,162.27	265.3 ± 587.11	604.63 ± 822.72
Non-america (*n* = 37)	38,906.21 ± 75,310.26	247.35 ± 455.21	83.72 ± 179.3	819.3 ± 983.15
*p*	0.395	0.248	**0.039**	0.262
**Country**				
USA (*n* = 60)	71,712.68 ± 191,240.08	929.26 ± 2,210.01	262.71 ± 593.19	603.87 ± 839.48
Other (*n* = 40)	38,505.75 ± 734,334.65	256.3 ± 461.49	101.22 ± 221.38	804.35 ± 951.29
*p*	0.439	0.436	0.066	0.361

**Note:**

Mann Whitney U test. USA, United States of America, Bold indicates statistical significance (*p* < 0.05).

## Discussion

This study evaluated the content, quality, reliability, and user interaction parameters for videos on post-COVID pain on YouTube, which has grown in popularity during the pandemic. We conducted our study on YouTube because it is the leading video-sharing platform. Although there have been several studies on YouTube videos about COVID-19 and long COVID-19, to the best of our knowledge, there has been no study on post-COVID pain, an important symptom of the disease that has a negative impact on the quality of life (Chan et al., 2021; Jacques et al., 2022; Kocyigit & Akyol, 2021). Considering that pain is a major cause of disability and can last for up to 11 weeks after infection, disrupts patients’ quality of life, and causes them to stay home during that period and search for disease-related information online, it is important to screen YouTube videos on post-COVID pain and evaluate their reliability and quality (Jacques et al., 2022, Şahin et al., 2021). The present study found that most of the videos had been uploaded in 2021, videos uploaded by academic sources were of higher quality and reliability, whereas videos uploaded by news channels were of lower quality and reliability. Overall, 74% of videos offered low-quality content based on their GQS scores, and only 6% contained sufficient data based on JAMA scores.

In this study, health-related websites (33%) and news channels (28%) were the most common sources of upload. In line with the results of this study, Parabhoi et al. (2021) and Szmuda et al. (2020) that evaluated YouTube videos on COVID-19 reported that news channels were the most common source of upload. Throughout the COVID-19 pandemic, news channels have shared developments, daily numbers of cases and deaths, and vaccination statistics in real time on television news channels, news websites, and YouTube. Users have used social media platforms to quickly access information and address their concerns on the internet, which can be explained by the fact that content originating from news channels was more common compared to other content types.

Studies in the literature point out that the sources of YouTube videos are diverse and the quality of videos varies with the source. A study by Azak et al. (2022) evaluated YouTube videos on COVID-19 in children and, in line with the results of our study, found that DISCERN scores were higher in videos uploaded by academic sources and lower in news videos; they found the difference to be statistically significant. Similarly, Li et al. (2020) evaluated YouTube videos on COVID-19 and found that, entertainment news videos had low reliability and quality than government/Professional videos, along with low DISCERN and JAMA scores. The results of our study are consistent with other studies in the literatüre. In addition, there are studies showing that videos on Personal protective equipment in COVID prepared by Public Health Institutions have high DISCERN and GQS results, while videos created by individuals have low scores (Gerundo et al., 2022). The present study showed that academic videos based on science offered high-quality and reliable content, yet videos uploaded by news channels provided inaccurate and misleading information to attract user attention and interaction. Low-quality videos offer content that lacks appropriate and adequate references, often fail to address the issue from different perspectives, and contain incomplete data. Such videos do not allow internet users to get answers to their questions or, through misleading information, cause them to adopt inappropriate attitudes. When searching for health-related information on the internet, users should aim to access better quality videos and should opt for videos that provide up-to-date and adequate resources. Academic sources and physicians should increase their presence on social media platforms by uploading up-to-date and accurate content.

YouTube is an interactive platform that allows users to provide simple and quick feedback by clicking on like buttons. Increased user parameters in videos attract more interaction, allowing the video to be viewed by more people. The present study demonstrated that news videos had more views, likes, and comments, whereas videos uploaded by academic sources and physicians had longer durations. Jacques et al. (2022) reported that videos on long COVID-19 that originated from TV-entertainment channels received more likes compared to videos uploaded by professional sources. Andika et al. (2021) evaluated YouTube videos related to COVID-19 and found that videos containing misleading information had statistically significantly higher numbers of likes and dislikes. This can be explained by the fact that internet users have limited knowledge of the criteria for selecting relevant videos, making it difficult to select quality videos. Another inference is that video user parameters are not indicative of video quality. Therefore, when deciding which videos to watch, users should check video sources rather than video parameters.

In terms of subject content, treatment-related subjects were the most common type of content found in the present study, with 79 (79%) videos. In line with our study, Parabhoi et al. (2021) examined YouTube videos on COVID-19 and found that most of the videos were about treatment-related issues. Another study by Baytaroglu & Sevgili (2021) that evaluated YouTube videos on peripheral artery disease found that most videos were about symptoms and treatment. In terms of the year of upload, there was a significant correlation between videos on diagnosis and the year of upload; videos on diagnosis decreased gradually over time. Considering that the most popular subjects during the COVID-19 pandemic changed over time, with prevention, treatment, and vaccination becoming the most popular, websites posted varying topics based on the popularity of subjects at a given time.

Initially, medical societies had refused to recognize long COVID as a disease (Rushforth et al., 2021). However, as survivors shared their health issues on social media, scientific authorities began to pay attention to these conditions and started to believe that the symptoms mentioned by survivors could be a sequela of the disease (Jacques et al., 2022). Furthermore, Callard & Perego (2021) stated that long COVID could be defined as “the first illness collectively created by patients finding one another through Twitter and other social media.” Symptoms of pain that persist after recovery from a life-threatening disease can affect physical and mental health. Rehabilitation services, psychological services, and medical treatments should be made available to patients (Sheehy, 2020). For most patients, routine medical care should continue at home, and telemedicine should be used to avoid disruptions in healthcare access (Eccleston et al., 2020). Given that persistent pain is likely to worsen depression and anxiety and, as a result, reduce the quality of life, it is clear that clinicians should pay closer attention to pain symptoms as one of the post-COVID manifestations and develop new treatment strategies (Şahin et al., 2021).

Like other studies investigating YouTube videos, our study has some limitations; for instance, we used information that was available at a specific point in time. YouTube is known to offer dynamic content and is a constantly evolving and changing social media platform. Similar studies conducted at different times may yield different results. The use of only English-language videos may also be considered a limitation. In addition, the process of video evaluation is subjective, although the two authors were blind to each other’s evaluations. One final limitation is that different search terms could have been used, which would have affected the results of the study.

## Conclusion

This study analyzed YouTube videos related to post-COVID pain. Three-quarters of the videos evaluated in our study were found to be of low quality and about 10% were of high quality. The reliability assessment found that only 6% of the videos contained completely sufficient data, that high-quality and reliable videos had longer durations, and that they were uploaded by academic sources and physicians. Videos produced by news channels were found to have more views, likes, comments, and shorter video durations but had low quality and low reliability. It can be argued that high-quality and reliable videos posted by academic sources and physicians on YouTube, a useful source of information, can help patients suffering from post-COVID pain. Users should choose videos on YouTube according to the source of the videos in order to access accurate and high-quality information, and they should not be affected by video parameters, such as the number of views, likes, and comments. Video producers who offer high-quality videos, including academic sources and physicians, should also be encouraged to create more online content. Allowing content supervised by a rated institution while sharing health-related information in digital environments may be more beneficial for public health. Considering that pain symptoms can persist after the disease and worsen the quality of life and COVID can cause new sequelae over time, it is clear that further well-designed studies are needed to examine painful conditions associated with COVID-19. New studies that evaluate videos in languages other than English and videos with a longer date range may have fewer limitations.

## Supplemental Information

10.7717/peerj.14089/supp-1 Supplemental Information 1 Raw Data.

## Additional Information and Declarations

### Data Availability

The following information was supplied regarding data availability: The raw measurements are available in a Supplemental File.

## References

[ref-1] Andika R, Kao CT, Williams C, Lee YJ, Al-Battah H, Alweis R (2021). YouTube as a source of information on the COVID-19 pandemic. Journal of Community Hospital Internal Medicine Perspectives.

[ref-2] Azak M, Şahin K, Korkmaz N, Yıldız S (2022). YouTube as a source of information about COVID-19 for children: content quality, reliability, and audience participation analysis. Journal of Pediatric Nursing.

[ref-3] Baytaroglu C, Sevgili E (2021). Characteristics of YouTube videos about peripheral artery disease during COVID-19 pandemic. Cureus.

[ref-4] Bernard A, Langille M, Hughes S, Rose C, Leddin D, Veldhuyzen van Zanten S (2007). A systematic review of patient inflammatory bowel disease information resources on the World Wide Web. The American Journal of Gastroenterology.

[ref-5] Callard F, Perego E (2021). How and why patients made Long COVID. Social Science & Medicine.

[ref-6] Chan C, Sounderajah V, Daniels E, Acharya A, Clarke J, Yalamanchili S, Normahani P, Markar S, Ashrafian H, Darzi A (2021). The reliability and quality of YouTube videos as a source of public health information regarding COVID-19 vaccination: cross-sectional study. JMIR Public Health and Surveillance.

[ref-7] Charnock D, Shepperd S, Needham G, Gann R (1999). DISCERN: an instrument for judging the quality of written consumer health information on treatment choices. Journal of Epidemiology and Community Health.

[ref-8] Chaveesuk S, Khalid B, Chaiyasoonthorn W (2021). Digital payment system innovations: a marketing perspective on intention and actual use in the retail sector. Innovative Marketing.

[ref-9] Eccleston C, Blyth FM, Dear BF, Fisher EA, Keefe FJ, Lynch ME, Palermo TM, Reid MC, Williams A (2020). Managing patients with chronic pain during the COVID-19 outbreak: considerations for the rapid introduction of remotely supported (eHealth) pain management services. Pain.

[ref-10] Ge H, Wang X, Yuan X, Xiao G, Wang C, Deng T, Yuan Q, Xiao X (2020). The epidemiology and clinical information about COVID-19. European Journal of Clinical Microbiology & Infectious Diseases: Official Publication of the European Society of Clinical Microbiology.

[ref-11] Gerundo G, Collà Ruvolo C, Puzone B, Califano G, La Rocca R, Parisi V, Capece M, Celentano G, Creta M, Rengo G, Leosco D, Abete P, Longo N, Mirone V, Ferrara N (2022). Personal protective equipment in Covid-19: evidence-based quality and analysis of YouTube videos after one year of pandemic. American Journal of Infection Control.

[ref-12] Greenhalgh T, Knight M, A’Court C, Buxton M, Husain L (2020). Management of post-acute COVID-19 in primary care. BMJ (Clinical Research Ed.).

[ref-13] Jacques ET, Basch CH, Park E, Kollia B, Barry E (2022). Long Haul COVID-19 videos on YouTube: implications for health communication. Journal of Community Health.

[ref-14] Kocyigit BF, Akyol A (2021). YouTube as a source of information on COVID-19 vaccination in rheumatic diseases. Rheumatology International.

[ref-15] Li HO, Bailey A, Huynh D, Chan J (2020). YouTube as a source of information on COVID-19: a pandemic of misinformation?. BMJ Global Health.

[ref-16] Muangmee C, Kot S, Meekaewkunchorn N, Kassakorn N, Khalid B (2021). Factors determining the behavioral intention of using food delivery apps during COVID-19 pandemics. Journal of Theoretical and Applied Electronic Commerce Research.

[ref-17] Nabavi N (2020). Long COVID: how to define it and how to manage it. BMJ (Clinical Research Ed.).

[ref-18] Onder ME, Zengin O (2021). YouTube as a source of information on gout: a quality analysis. Rheumatology International.

[ref-19] Parabhoi L, Sahu RR, Dewey RS, Verma MK, Kumar Seth A, Parabhoi D (2021). YouTube as a source of information during the COVID-19 pandemic: a content analysis of YouTube videos published during January to March 2020. BMC Medical Informatics and Decision Making.

[ref-20] Rittberg R, Dissanayake T, Katz SJ (2016). A qualitative analysis of methotrexate self-injection education videos on YouTube. Clinical Rheumatology.

[ref-21] Rushforth A, Ladds E, Wieringa S, Taylor S, Husain L, Greenhalgh T (2021). Long COVID – The illness narratives. Social Science & Medicine.

[ref-28] Şahin T, Ayyildiz A, Gencer-Atalay K, Akgün C, Özdemir HM, Kuran B (2021). Pain symptoms in COVID-19. American Journal of Physical Medicine & Rehabilitation.

[ref-22] Sheehy LM (2020). Considerations for postacute rehabilitation for survivors of COVID-19. JMIR Public Health and Surveillance.

[ref-23] Silberg WM, Lundberg GD, Musacchio RA (1997). Assessing, controlling, and assuring the quality of medical information on the internet: caveant lector et viewor--let the reader and viewer beware. JAMA.

[ref-24] Szmuda T, Syed MT, Singh A, Ali S, Özdemir C, Słoniewski P (2020). YouTube as a source of patient information for Coronavirus Disease (COVID-19): a content-quality and audience engagement analysis. Reviews in Medical Virology.

[ref-25] Tang D, Comish P, Kang R (2020). The hallmarks of COVID-19 disease. PLOS Pathogens.

[ref-26] Tolu S, Yurdakul OV, Basaran B, Rezvani A (2018). English-language videos on YouTube as a source of information on self-administer subcutaneous anti-tumour necrosis factor agent injections. Rheumatology International.

[ref-27] WHO Team (2022). Weekly epidemiological update on COVID-19–20 April 2022. https://www.who.int/publications/m/item/weekly-epidemiological-update-on-covid-19---20-april-2022.

